# Birch (*Betula pubescens* Ehrh.) Encroachment Alters Contribution of Plant Functional Groups to Ecosystem Carbon Cycling in a Rewetted Bog

**DOI:** 10.1002/ece3.70745

**Published:** 2024-12-18

**Authors:** Carla Welpelo, Maren Dubbert, Bärbel Tiemeyer, Alexander Knohl, Arndt Piayda

**Affiliations:** ^1^ Thünen Institute of Climate‐Smart Agriculture Braunschweig Germany; ^2^ Leibniz Centre for Agricultural Landscape Research (ZALF) Müncheberg Germany; ^3^ Bioclimatology University Göttingen Göttingen Germany

**Keywords:** bog restoration, C balance, CO_2_, GHG balance, peatlands, rewetting, tree encroachment

## Abstract

Rewetted bogs with high water levels (WL) and mire‐specific vegetation are crucial carbon (C) sinks, but their function might be threatened by tree encroachment, a phenomenon widespread in the northern hemisphere that often coincides with low WL. This might impact C cycling both at the ecosystem and microform scale in multiple ways, but so far, data are lacking. We established two sites in the same former peat extraction area, one showing permanently high WL and mire‐specific vegetation (open site, OS), while the other one has more fluctuating WL and a dense birch ( *Betula pubescens* Ehrh.) population (tree site, TS). We measured the carbon dioxide (CO_2_) exchange at ecosystem (eddy covariance) and plot scale (chamber measurements) for 1 year to clarify the differences between the sites and the impact of birch encroachment on the contribution of the different bog‐specific microforms and the trees to the ecosystem's CO_2_ balance. Overall, the OS had a CO_2_ balance of −262.4 ± 7.8 g CO_2_‐C m^−2^ year^−1^ indicating CO_2_ uptake, while the TS was close to neutral (−28 ± 5.1 g CO_2_‐C m^−2^ year^−1^). The smaller uptake at the TS was caused by higher (151%) ecosystem respiration, while gross primary production was 14% higher. However, the microform contributions to C uptake strongly differed: At the OS, both hummocks and hollows showed net uptake, while at the TS, most C (52%) was assimilated by the birches and the understory was a net CO_2_ source. This indicates a loss of peat C from the TS, while the successfully rewetted site was accumulating new peat. Accounting for plot‐scale CH_4_ fluxes, both sites were a weak source of greenhouse gases, but a distinctly stronger C sink occurred at the OS. Our data show the possibility of increasing C removal from the atmosphere by full rewetting and the establishment of mire‐specific vegetation.

## Graphical abstract

Rewetted bogs with high water levels (WL) and mire‐specific vegetation function as important carbon sinks, but tree encroachment, often associated with low WL, may threaten this. Our study compared CO_2_ exchange at two bog sites—one with high WL and mire‐specific vegetation (open site, OS) and another with fluctuating WL and dense birch encroachment (tree site, TS). Results showed that the OS had a strong CO_2_ uptake (−262.4 g CO_2_‐C m^2^ year^−1^), while the TS was nearly neutral (−28 g CO_2_‐C m^2^ year^−1^), indicating peat carbon loss at the TS despite similar levels of gross primary production.

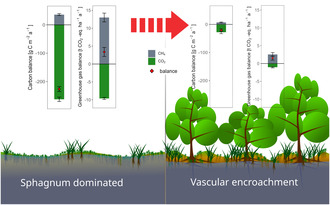

## 1 Introduction

Peatlands are important carbon (C) pools, storing up to 30% of the terrestrial organic C while covering only around 3.8% of the earth's surface (UNEP 2022). They are under constant pressure due to climate change and increasing anthropogenic activities. Peat extraction and drainage for agriculture and forestry impair or reverse the C sink function of natural mires. Drained peatlands are net sources of greenhouse gases (GHG), mainly carbon dioxide (CO_2_), and emit 1.91 (0.21–3.38) Gt CO_2_‐eq. year^−1^ globally (Leifeld and Menichetti 2018). In Europe, around 53% of peatlands remain undegraded, and in Germany only around 10% (UNEP 2022). Peatland rewetting is one of the prime options to remove atmospheric CO_2_, as rewetted peatlands have the potential to act as a long‐term C sink despite their low land share (Günther et al. 2020; IPCC 2022; Yu et al. 2010).

For decades, peatland rewetting primary aim at restoring mire‐specific hydrology and vegetation (Hammerich et al. 2022), protecting endangered species and ultimately increasing biodiversity in rare habitats (Andersen et al. 2017; Tanneberger et al. 2021). With growing consciousness about climate change, the focus on rewetting has shifted to the conversion of degraded peatlands from CO_2_ sources to C neutrality or even C sinks. High water levels (WL) are necessary to both limit emissions from peat mineralisation (Tiemeyer et al. 2020) and to ensure the growth of mire‐specific vegetation (Hammerich et al. 2022), which determines the C accumulation rate and thus, C sink strength of these ecosystems (Loisel and Yu 2013; Mathijssen et al. 2019). The proven concept of peatland rewetting is widely proposed as an acknowledged and necessary measure to meet (inter‐)national emission reduction targets (BMUV 2022; IPCC 2022; Tanneberger et al. 2021).

The natural vegetation of temperate and northern ombrogenic bogs is dominated by *Sphagnum* mosses, which are the main peat‐forming plant genus (Clymo and Hayward 1982). They are accompanied by graminoids such as *Eriophorum* ssp. and scattered shrubs and trees. In bogs, vegetation is often distributed patterned on microforms, broadly described as hummocks and hollows. Hollows provide wetter habitats and are dominated by correspondent *Sphagnum* species. Hummocks, with an elevated surface and therefore lower WL, have higher soil temperatures, increased nutrient availability and show—besides adapted *Sphagnum* species—a higher density of vascular plants (Holmgren et al. 2015).

The success of peatland rewetting, especially in bogs, is determined by a combination of environmental conditions, individual site characteristics and an adequate choice of technical measures (Rowland et al. 2021). Consequently, it is not always completely successful: Changing climatic conditions such as longer summer droughts and higher temperatures as well as water losses due to unrecognised drainage, permeable subsoil or insufficient rewetting due to land use conflicts can hamper the rewetting success. This can lead to unwanted effects such as tree encroachment (Heijmans et al. 2013; Jagodzinski et al. 2017). Increased nutrient depositions from surrounding agricultural areas is also suspected to change vegetation composition in bogs (Breeuwer et al. 2010; Juutinen, Bubier, and Moore 2010; Tomassen et al. 2003). An encroachment by trees (especially *Betula*) and an accompanied higher density of graminoids in the understorey have been registered over the northern hemisphere in recent decades (Beauregard, Lavoie, and Pellerin 2020; Berg et al. 2009; Dyderski, Gdula, and Jagodziński 2015; Favreau, Pellerin, and Poulin 2019; Heijmans et al. 2013; Jagodzinski et al. 2017; Lanta and Hazuková 2005).

Tree encroachment has a strong impact on bog conditions: Dense root networks change water and oxygen availability, nutrient supply and concentrations of dissolved organic carbon (DOC) within the peat (Ratcliffe et al. 2017; Wertebach et al. 2016; Zeh et al. 2019). This is consequently effecting peat structure and decomposition rate (Gogo et al. 2011). Aboveground, tree encroachment results in increased shading, altered litter volume and quality along with lower wind speed (Zhang et al. 2019). This, in turn, affects vegetation composition and richness, leading to a changing ecosystem association of the vegetation (Favreau, Pellerin, and Poulin 2019; Ratcliffe et al. 2017). Due to drier conditions, trees prefer germination on hummocks (Holmgren et al. 2015). Tree encroachment in combination with drier ecohydrological conditions can lead to an even more hummock‐dominated surface pattern and vice versa (Eppinga et al. 2009; Harris, Roulet, and Moore 2020; Malhotra et al. 2016). This can decrease *Sphagnum* density (Malhotra et al. 2016; Pellerin, Lavoie, and Talbot 2021), generally seen as adverse. The overall impact on the net ecosystem exchange (NEE) remains yet unknown.

Vegetation composition is a major factor regarding the C balance of bogs. *Sphagnum* species photosynthesise all year when not covered by snow (Robroek et al. 2009; Walker et al. 2017). Deciduous *Betula* trees have a higher C uptake per leaf area than graminoids or bryophytes, but only during the vegetation period (Heskel et al. 2013; Korrensalo et al. 2017). Even though only part of the C assimilated by *Betula* is stored in relatively stable biomass, the investment in wood and roots is high, especially in younger stands (around 77% of assimilated C) (Uri et al. 2017). Under dry conditions, the wood is not stored as peat and the assimilated C is released back into the atmosphere after decades to centuries. In contrast, moss biomass can, under anaerobic, bog‐typical conditions, be permanently stored as peat, that is, contribute to long‐term bog growth (Succow and Joosten 2012).

Existing data report the NEE of naturally wooded bogs (Strilesky and Humphreys 2012; Syed et al. 2006) or bogs drained for forestry (Hommeltenberg et al. 2014; Ojanen et al. 2019), but hardly anything is known about the impact of the change towards tree domination on CO_2_ emissions and C sequestration rates. The encroachment of trees on bogs is a widely observed process which remains yet unstoppable with decades of restoration efforts at stake. The feedback loop in the changing climate system is still unknown, preventing the implementation of changes in emission inventories.

In order to gain process understanding, this study investigates the contribution of different microforms as well as trees to the overall ecosystem CO_2_ balance. We combine fluxes obtained from chamber measurement with data from two Eddy covariance (EC) towers from the same observation period on two neighbouring, but contrasting sites. Both sites are in the same former peat extraction area and were rewetted simultaneously, one of them with mire‐specific vegetation and permanent WL and one exhibiting a dense, encroaching birch population and fluctuating, lower WL. We conducted chamber measurements at different microforms and the birch branches, enabling the disentanglement of different plant functional group contributions to total CO_2_ uptake—and release. Methane (CH_4_) was also measured in parallel to the CO_2_ measurements, allowing GHG and C balances to be calculated.

The main research questions were as follows:

1. How do both sites differ in NEE?
2. How do birch encroachment and lower WL alter CO_2_ fluxes at the microform level? What is the total contribution of the birches to ecosystem CO_2_ fluxes?
3. What is the impact of the differences regarding WL and vegetation on the GHG and C balance?

## 2 Material and Methods

### 2.1 Investigation Area

**FIGURE 1:**
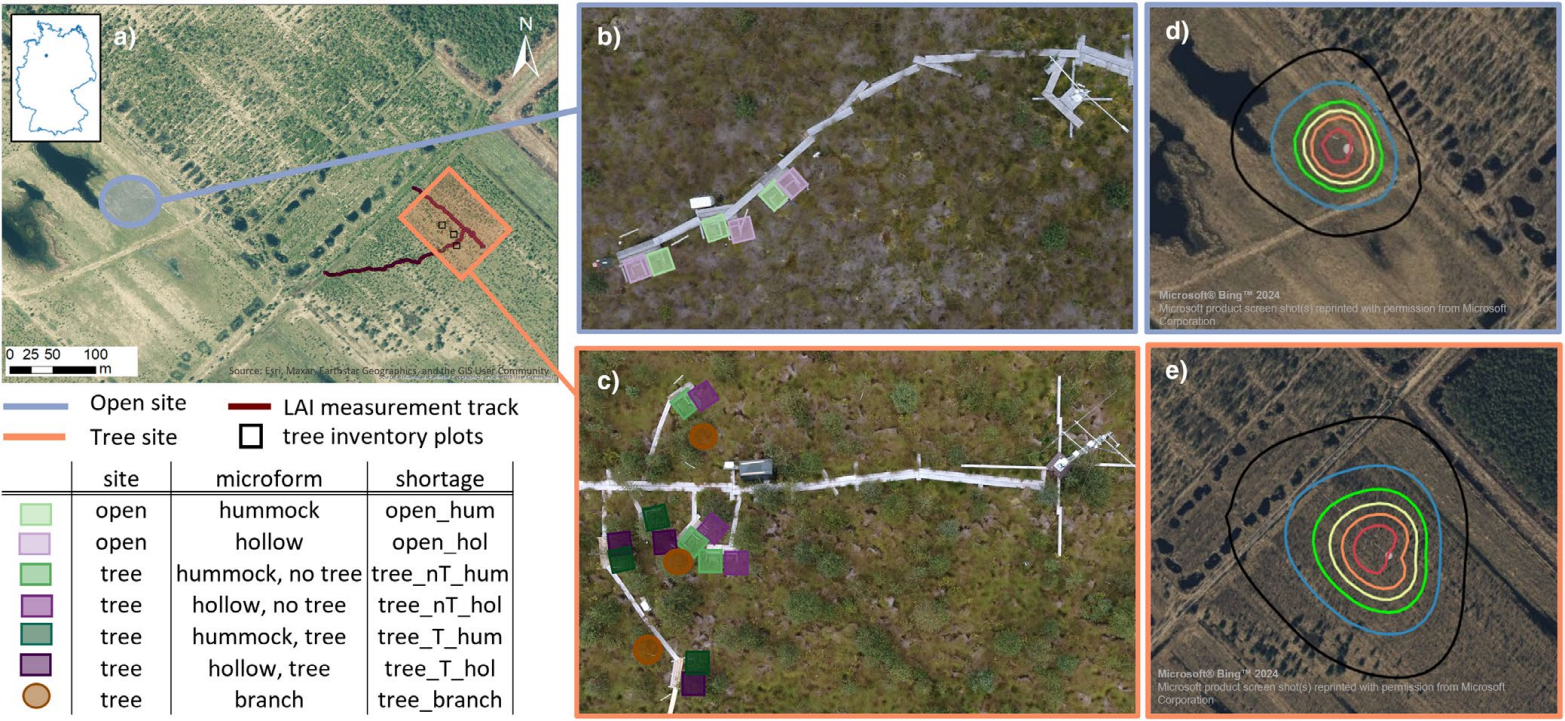
(a) Overview of the investigation area. (b) Open site with the Eddy covariance (EC) tower (upper right) and the three replicates of the microform plots (hummock and hollows). (c) Tree site with three replicates of microform plots and the plots for branch measurements. The EC tower is visible on the right site. (d, e) EC footprints of open site (d) and tree site (e). Colours mark the 20% (red), 40% (orange), 50% (yellow), 60% (green), 70% (blue) and 80% (black) contribution to the flux data.

The investigation sites are located in the nature conservation area ‘Weißer Graben’ (502 ha) in Northwest Germany (52°42′02″ N 9°22′05″ E), which is part of the bog complex Lichtenmoor (2220 ha). Mean annual precipitation is 731 mm and the mean annual temperature is 9.9°C (reference period 1991–2020, German Climate Service, Station Nienburg (Weser)). The rewetting commenced in 1984 following the cessation of peat extraction. The two sites were set up in two different parcels, typically separated with dams to facilitate water retention (Figure 1a). One of the sites is located in the centre of the nature conservation area and has a high cover of *Sphagnum* mosses (mainly *S. cuspidatum* Ehrh. ex Hoffm.) and *Eriophorum* (mainly *E. vaginatum* L.). Plot‐wise vegetation data are available in Table A4. This site represents mire‐specific vegetation (based on Hammerich et al. (2022), although hummock species are still largely absent), with a low density of trees and shrubs and a constantly high WL (*open site*, Figure 1a).

The second site is around 400 m eastwards, closer to the edge of the rewetted area. The WL is more variable, which is suspected to be caused by an ongoing unintentional drainage due to a deeply drained adjacent grassland at the southeast border of the rewetted area and an ineffectively blocked ditch leaving the area at the northeast edge. Vegetation is dominated by a dense birch population (mainly *Betula pubescens* ) with *Eriophorum vaginatum* in the understory and a much lower *Sphagnum* density (mainly *S. cuspidatum* ). The maximum age of the birches is around 10 years, with a high share of younger trees (*tree site*, Figure 1b).

The prevailing soil type at the open site was classified as an Ombric Fibric Floatic Histosol (WRB 2022) with a mean peat thickness of around 151 cm and a rooting depth of up to 92 cm. Almost undecomposed peat of the upper soil horizons with around 56 cm of accumulated organic material since rewetting reflects the water‐saturated conditions after peat extraction (H2 to H4 according to von Post (1924)). The lower soil horizons showed moderately to very highly decomposed peat (> 68 cm: H5 to H8).

At the tree site, only the first 28 cm had weakly decomposed organic material which accumulated after rewetting (H2 to H4), followed by highly decomposed peat in deeper horizons (H7 to H9 according to von Post (1924)). Accordingly, it was classified as Ombric Sapric Histosol (WRB 2022) with an average peat depth of around 100 cm. Rooting depth was very inhomogeneous, with living roots beyond the first meter. Even if the C contents were similar, the carbon‐to‐nitrogen (C:N) ratio in the upper horizon was much higher at the open site (open site: 61 in 0–11 cm; tree site: 42 in 0–30 cm) due to higher nitrogen contents of the peat. Tree litter C:N ratio was 41 ± 3, which is similar to peat C:N ratio at the tree site. Furthermore, plant‐available phosphorus in the upper horizon is higher at the tree site (details on peat properties in Table A1).

Both sites are characterised by a distinctive surface structure consisting of hummocks and hollows. Hollows are lower, do consequently have higher WL and are dominated by *Sphagnum ssp*. Hummocks are more elevated, therefore drier and mainly vegetated with *Eriophorum vaginatum* . At the open site, plots are divided into hummocks and hollows (open_hum and open_hol). At the tree site, plots are further divided into hummocks and hollows close to trees (tree_T_hum and tree_T_hol) and more distant to trees (tree_nT_hum and tree_nT_hol). Additionally, the birch branches at the tree site are named tree_branch.

### 2.2 Environmental Data

The areal contribution of each microform (hummock, hollow, tree/no tree) was determined for both sites at each cm of 5 (open site) or 10 (tree site) 10‐m‐long transects crossing the EC footprint. The trees were divided into three size classes and a maximum distance was determined for each class (0.5, 1 and 1.5 m) to subdivide the transects into nT and T areas. Details can be found in Welpelo et al. (2024) and Table A2.

WL were measured in observation wells on both sites using three to four absolute pressure transducers (Van Essen Instruments, Micro‐Diver), distributed in the footprint area. Pressure was recorded every 30 min and compensated with ambient air pressure from a barometer (Van Essen Instruments, Micro‐Baro), placed at one of the EC towers. In addition, tensiometers (UMS GmbH, T8) were used to measure the matric potential inside a hummock and a hollow at each site and one additional tensiometer at the tree site in a hollow next to a tree (around 5 to 10 cm depth).

To measure the development of tree heights and density, birch inventories were conducted in late summer of both years (2020 and 2021), measuring tree height, species, stem circumference and crown diameter of each tree in three inventory plots (100 m^2^ each, Figure 1a). Seedlings below 50 cm were only counted, but not measured.

Leaf area index (LAI) of the trees was measured biweekly in the vegetation period 2021 (Plant Canopy Analyzer (LI‐COR, LAI‐2200C)) on a transect (back and forth looking at different cardinal directions) spanning 280 m across the footprint comprising 50 single observations (Figure 1a). The tree LAI includes *Betula* and *Pinus* trees, but the share of pine was ignored due to the small total number of individuals (7%). As the LAI‐2200C is measuring plant area index, the LAI might have been overestimated to a certain amount.

### 2.3 Measurement of CO_2_ Fluxes

The EC method was employed to obtain ecosystem‐scale fluxes, while the chamber method was used on a campaign basis to differentiate these fluxes into the contribution of microforms and trees. If not mentioned otherwise, all analyses were conducted using R software version 4.4.1 (R Core Team 2024). For all reported fluxes, the atmospheric sign convention is used, which defines negative fluxes as uptake by the ecosystem and positive fluxes as emissions to the atmosphere.

#### 2.3.1 Eddy Covariance

Before the field measurements started, both analysers ran simultaneously to ensure comparable results. Even though the systems are slightly different, it has been shown that the results regarding CO_2_ fluxes are comparable (Goodrich et al. 2016; Haslwanter, Hammerle, and Wohlfahrt 2009).

An EC tower with the corresponding bio‐meteorological sensors was installed at both sites in mid‐2020. The data collection for this publication took place from November 2020 to October 2021, as chamber measurements are available for the same period (see below). Both systems were operated with solar power, which led to small data gaps in winter. At the Open site, an open path gas analyser (OP) (LI‐COR, LI‐7500A) was mounted at 2.5 m height. It was installed at an approximately 45° angle to prevent the accumulation of water, snow or dirt on the optical path. Next to the gas analyser, an anemometer (Gill, R3‐50) was placed with a northward separation of approximately 20 cm. The anemometer and the gas analyser were logging in 20 Hz intervals. Biometeorological data were logged (Campbell Scientific, CR1000) in 1‐min intervals. Sensors for radiation components (incoming and outgoing longwave and shortwave radiation (Kipp & Zonen, CNR4) and up‐ and downwards photosynthetically activating radiation (PAR) (LI‐COR, Li‐190R)) were mounted on an approximately 1.5‐m‐long, south‐facing boom at 2 m height. Soil temperature (*T*_soil_) was measured at 5, 15 and 30 cm depths next to the tower (Campbell Scientific, 108 Temperature Probe). Relative humidity (RH) and air temperature were measured at 2 m height at the tower (Vaisala, HUMICAP HMP155).

At the tree site, an enclosed path gas analyser (CP) (LI‐COR, Li‐7200) was installed at approximately 7.5 m height. The analyser was supplied with ambient air through a heated intake tube (78 cm) with a flow rate of 15 L per minute and the opening was installed directly below the anemometer (around 2 cm downward separation). The anemometer was the same model as at the open site and data collection was set to 20 Hz. Bio‐meteorological data were logged by the Data Acquisition Module (LI‐COR, Data Acquisition System). The same radiation sensors as at the open site were employed and, because of the higher tower, mounted on a 3‐m‐long, southward‐facing boom at 7 m height. RH, ambient pressure and air temperature were measured (METER Group, ATMOS 14) at around 5 m height, and *T*_soil_ was measured at 5, 15 and 30 cm depth next to the tower. Additionally, three below tree canopy PAR sensors (LI‐COR, Li‐190R) were installed on the south site below randomly selected *Betula* trees close to the EC.

Precipitation data were taken from the closest weather station of the Germany Climate Service for both sites (DWD; Station Nienburg (Weser), 12 km).

The software EddyPro (LI‐COR, version 7.0.9) was used for flux calculations. Settings are collected in Table A3. Data were further filtered and tested for outliers following Papale et al. (2006). The R package REddyProc (Wutzler et al. 2022) was used to calculate friction velocity thresholds and to conduct gap filling and flux partitioning. The final footprints (Figure 1d,e) were calculated using the model presented by Kljun et al. (2015) and the available R code. Further information about the processing of the EC data can be found in the Appendix (Section A.2.1).

#### 2.3.2 Chamber Measurements

Manual dynamic closed chambers (Fiedler et al. 2022; Livingston and Hutchinson 1995) were used to measure CO_2_ exchange from 19 September 2020 to 20 October 2021. Measurement campaigns took place every 3 (March—October) to 4 (October—February) weeks, starting before sunrise and continuing throughout the day, to cover a wide range of radiation and *T*_soil_. Each campaign lasted 3 days, one measuring at the open site and two days at the tree site. Opaque (PVC) and transparent (transparent polycarbonate) chambers were used in the same plots. At least four measurements of each kind were aimed for per plot and campaign, distributed over the environmental conditions of the day. One measurement lasted between 120 and 180 s.

A Cavity Ring‐Down Spectroscopy Gas Analyzer (GasScouterTM G4301, Picarro) was used for the gas measurements. The GasScouter was combined with a data logger (HOBO UX120‐006M until May 2021, afterwards LabJack U3) to record temperatures inside and outside the chamber and, in the case of measurements with transparent chambers, PAR (LI‐COR, Li‐190R) outside the chamber. PAR was expected to be reduced by 5% inside the chamber and was consequently corrected for the calculations.

Plots were set up on microform pairs (hummocks/hollows) at each site inside the footprint of the EC system. Each variant had three replicates. At each plot, a PVC frame, on which the chamber was tightened with clamps, was firmly installed in the peat (around 10 cm). The plots were accessible by boardwalks on poles to prevent disturbance. *T*_soil_ was measured in 2 and 10 cm depth of the hummocks and by tensiometer in around 5–10 cm depth in the hollows.

In addition, cylindric branch chambers (*h* = 40 cm, *r* = 9.5 cm, transparent) were used to measure the gas exchange of birch branches. A fabric shading was used for the opaque measurements at branch chambers, with dark fabric inside and white fabric outside to prevent heating. The number of leaves inside each branch chamber was counted at each campaign. To calculate the leaf area inside, a leaf sample campaign took place in the summer of 2021. For this purpose, three branches of five trees were sampled and the leaves were scanned. The mean area per leaf was determined and used to calculate the leaf area inside each chamber at each campaign from the number of leaves counted. This leaf area was used as the source area for the flux calculation.

Simultaneously with each CO_2_ measurement, CH_4_ fluxes were measured. The annual sums were modelled using soil temperature and WL and scaled up to the ecosystem level based on the spatial distribution of microforms (Welpelo et al. 2024).

In total, 7 variants (5 at the tree site and 2 at the open site) including 21 plots were set up. Looking at the number of available CO_2_ fluxes used for the quality check and partitioning the quantity for each microform is between 165 and 187 (dark), respectively, 185–217 (transparent), which indicates a balanced database. Due to defoliation of the trees, branch measurements are fewer in total (102 (dark), respectively, 116 (transparent)). All available fluxes were used for the pairing with EC data (Section 3.3).

Further data processing and the calculation of annual balances were done following commonly used approaches. Fluxes were calculated using a linear regression inside a moving window. Minimal detectable fluxes were calculated according to Fiedler et al. (2022) and set to zero, illogical high morning fluxes were removed by chosen criteria. Daily fluxes were calculated as described in Leiber‐Sauheitl et al. (2014) and Oestmann et al. (2022). Fluxes were modelled for the period from 1 November 2020 to 31 October 2021 (hydrological year) to obtain daily NEE, GPP and *R*_eco_ and apply them in the source contribution. More detailed information about raw flux processing and flux calculation can be found in the Appendix (Section A.2.2).

For the calculation of the GHG balance, the emission factor of 27 was used to calculate the global warming potential (100 years horizon) for CH_4_ according to IPCC (2022). As the bog had never experienced agricultural land use and thus fertilisation and is fully vegetated, N_2_O emissions were expected to be negligible.

### 2.4 Comparison of Flux Measuring Methods and Source Contribution

To enable the partitioning of EC fluxes with chamber flux data, a correlation of both flux observation methods is mandatory. Therefore, chamber measurements were tested for comparability with the EC measurements.

To assess this, pairs of chamber fluxes were built for NEE, *R*_eco_ and GPP on certain conditions (Table 1). The aim was to add up the fluxes based on the areal share and to obtain an ecosystem flux that can be compared with the flux from EC. GPP of chamber measurements was calculated by subtracting the closest *R*_eco_ flux from each NEE flux from the same plot with a maximum time difference of 2 h. If no *R*_eco_ flux was available, no GPP was calculated. Each flux pair consists of a hummock and hollow observation—one pair at the open site and two pairs at the tree site (consisting of hummock and hollow observations close to trees (T) and a pair distant to trees (nT)). One pair was selected by smallest PAR difference (NEE, GPP) or smallest time difference (*R*_eco_). The same criteria were used to combine both pairs of the tree site. For the vegetation period (May to October), branch fluxes were additionally included. This way groups were formed consisting of two measurements at the open site and four to five measurements at the tree site.

**TABLE 1:** Thresholds and requirements for the pairing of chamber and EC fluxes.

Parameter	Threshold	Used for
PAR threshold	150 W m^−2^	NEE, GPP
ΔPAR	20%	NEE, GPP
Δ*T*_air_	2°C	NEE, GPP
Δ*T*_soil_	2°C	*R*_eco_
Tolerance for chamber pairing	90 min	NEE, GPP, *R*_eco_
Tolerance for EC pairing	4 days	NEE, GPP, *R*_eco_
Tolerance in daytime for EC pairing	2 h	NEE, GPP, *R*_eco_

Each flux was multiplied by the individual areal contribution of the respective microform and summed up to yield ecosystem‐scale fluxes. For the birch fluxes, the current LAI was used and added.

These flux sums were then correlated against EC fluxes (only original data with QC = 0). Pairing with EC data was conducted by the same PAR and time criteria as used for the chamber fluxes; conditions are shown in Table 1. An overview of the ratio of ecosystem fluxes derived by chamber measurements to those derived from EC and the number of the used fluxes is shown in Figure A2. Fluxes were tested for linearity using the rainbow test from the ‘lmtest’ package (Hothorn et al. 2022).

To calculate the contribution of each microform and the birches to GPP, *R*_eco_ and NEE, a method was developed that partitions daily fluxes of the EC system using the modelled balances. First, daily fluxes of *R*_eco_ and GPP derived from chamber measurements were used to split EC data into single proportionate contributions of the microforms.

Therefore, the fluxes were multiplied with the areal share of the respective microform or the current tree LAI to gain source flux per area. Then, daily flux sums were calculated as the sum of all single fluxes for each site to subsequently determine the relative flux contribution of each microform and the birch trees. The relative contributions were then multiplied with the daily GPP, respectively, *R*_eco_ fluxes derived from EC data. To obtain partitioned NEE fluxes, *R*_eco_ was subtracted from GPP for each source.

This way, the contribution of the microforms and the birches to the total ecosystem flux could be estimated on a daily basis and added up to an annual balance.

## 3 Results

### 3.1 Environmental Parameters and EC Data

**FIGURE 2:**
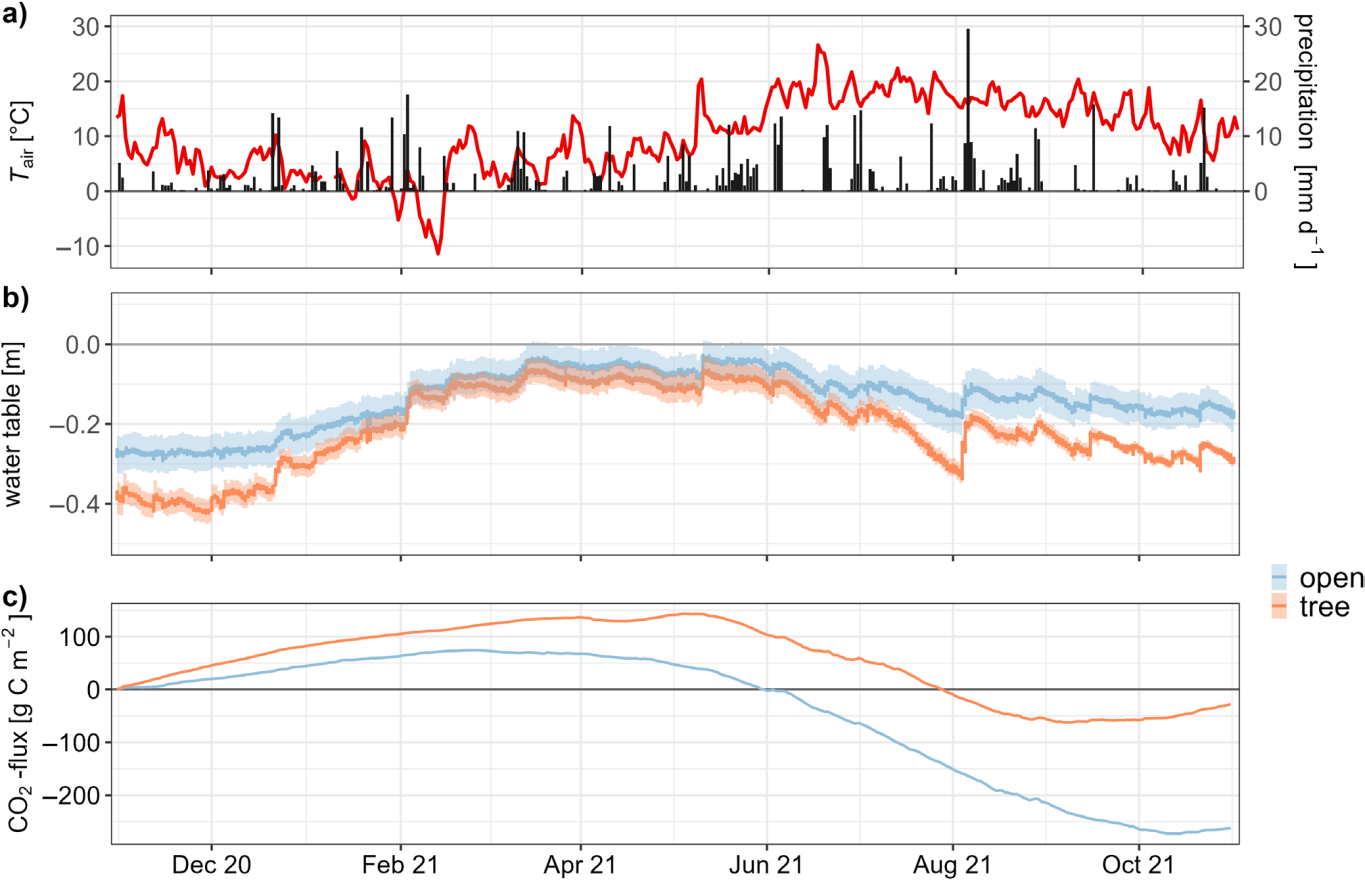
Environmental conditions and cumulative CO_2_ exchange during the measurement period (November 2020—October 2021). (a) Daily precipitation (Nienburg (Weser), 12 km) and mean daily temperature (*T*_air_) at the sites, (b) daily mean water level of both sites and (c) cumulative daily CO_2_ exchange of both sites.

Annual precipitation during the measurement period was 679 mm. That is lower than the long‐term annual mean but higher than in the preceding 3 years. There were precipitation events throughout the year and rainfall was particularly heavy in the summer period (Figure 2a). Mean annual temperature at the site was 9.9°C, with the daily minimum in February (−11.4°C) and the maximum in June (26.6°C). The data fit the long‐term annual temperature at the closest weather station.

WL follow the frequent precipitation events; that is, WL at the two sites were similar and around the surface during the wet spring of 2021. Both in autumn, winter and summer, the WL at the tree site was lower than at the open site (Figure 2b). Annual mean WL were −0.21 m ± 0.10 m at the tree site and −0.14 m ± 0.07 m at the open site, respectively.

**FIGURE A1:**
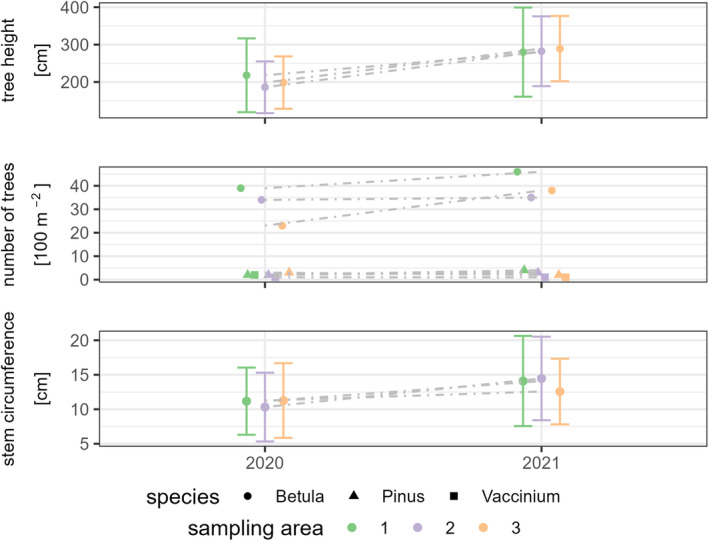
Development of the birch population over the investigation period on three sampling areas (10 m × 10 m). The inventories were carried out in June 2020 and August 2021.

Median height of birch trees was 2 m with maximum tree height reaching over 4 m at the beginning of the measurement period. Mean birch density was 32 trees on 100 m^2^ (3200 trees ha^−1^). At the end of the measurement period, the mean number of birches had risen to 40 on 100 m^2^ (4000 trees ha^−1^), the mean height to 2.8 m and the maximum tree height to 5.3 m (details in Figure A1).

Cumulative CO_2_ fluxes (NEE) derived by EC are shown in Figure 2c. During winter, both sites were net sources of CO_2_ (positive slope), with the tree site showing higher emissions. At the open site, a slight uptake began already in March (negative slope) and the uptake became stronger (steeper slope) in summer, lasting until mid‐October. In contrast, the tree site showed a clear net uptake only from May until September. That is mainly the period when birches have active leaves. In total, the tree site was a small sink, close to neutral and the open site was a strong net sink of CO_2_ in the measurement year (−28 ± 5.1 g CO_2_‐C m^−2^ year^−1^ and –262.4 ± 7.8 g CO_2_‐C m^−2^ year^−1^, respectively) (Table 3). Differences emerging by different gas analysers or by different tower heights cannot be taken into account and might lead to higher uncertainties in the results. However, as the differences in the CO_2_ balances are pronounced, this does not alter the overall findings.

### 3.2 Chamber Fluxes

**FIGURE 3:**
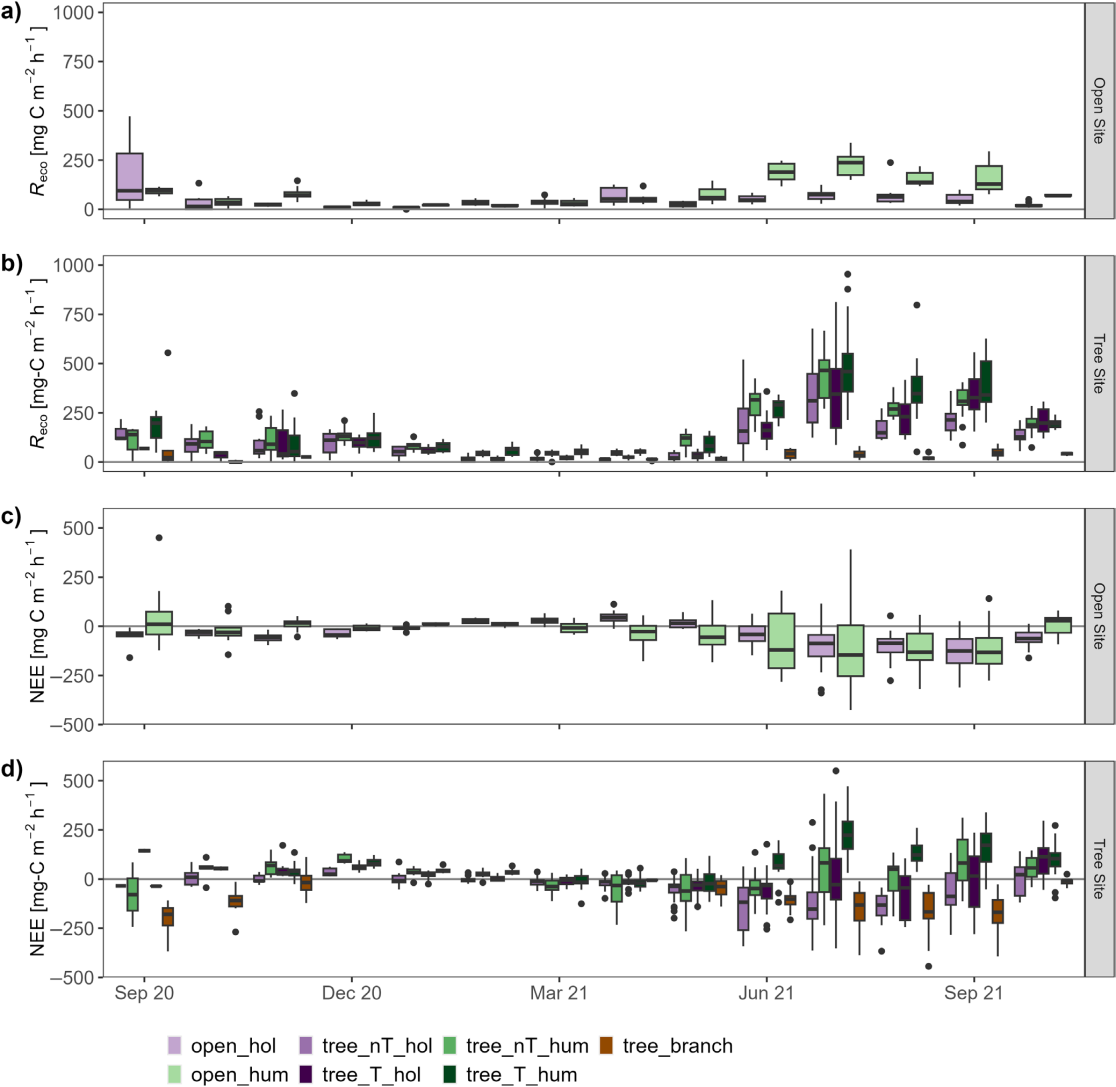
Boxplots of all available chamber fluxes for each microform and the branches, separated by month. Branch measurements are reported per leaf area (m^2^). Ecosystem respiration (*R*_eco_) was measured with opaque chambers at (a) open site and (b) tree site; net ecosystem exchange (NEE) was measured with transparent chambers at (c) open site and (d) tree site. The boxes represent the 25th and 75th percentile and the line in the box is the median. Whiskers are drawn until the last data point in 1.5 × interquartile range is reached. Points are data outside of this range. ‘hum’ stands for hummock plots, ‘hol’ for hollows, ‘nT’ means that there is no tree in direct proximity and ‘T’ stands for direct proximity to trees.

The magnitude of *R*_eco_ fluxes was higher in summer than in winter on both sites. By far the highest median for *R*_eco_ at the open site occurred in July (open_hum), while most microforms at the tree site regularly experience even higher rates of CO_2_ release in the summer months. The respiration of the trees was much lower per leaf area (Figure 3b). Highest single fluxes were generated at the tree site.

Variability of NEE measurements is high for both sites, as all different PAR and temperature conditions occurring during the campaigns of 1 month are grouped in one boxplot. At the open site, fluxes from hummocks were more variable than from hollows, which showed a net uptake in all seasons but during spring. At the tree site, all microforms were net CO_2_ sources in winter, until spring begins and a median around zero was observed. In summer, the hummocks were mostly releasing CO_2_, while hollows both distant to trees and—less consistently—close to a tree indicated small net uptakes. The branches were clear net sinks throughout the vegetation period (Figure 3d). Highest uptake rates in the summer months were measured at the branches and the hummocks at the open site.

### 3.3 Comparison of Flux Measuring Methods

Due to the larger number of microforms and the additional tree measurements, the number of groups which could be generated out of chamber measurements (described in Section 2.4) is clearly lower at the tree site in all cases (around 50%).

**FIGURE A2:**
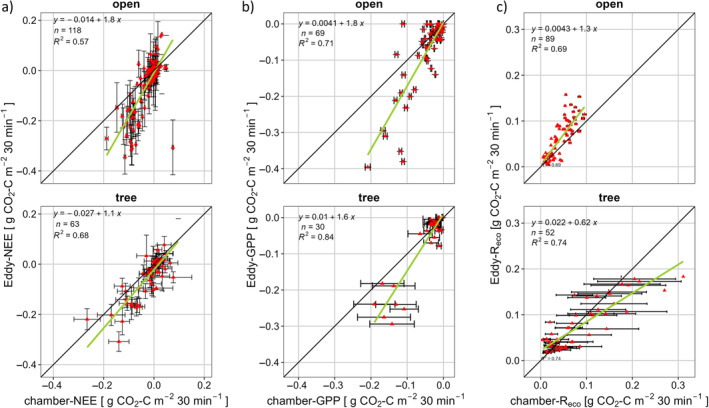
Full chamber fluxes and paired EC fluxes of both sites for NEE (a), GPP (b) and *R*_eco_ (c). Regression equation, *R* ^2^ and number of fluxes is given in the plot for both sites.

With coefficients of determination 0.57 and 0.68 for NEE (open and tree site, respectively) between chamber and EC measurements (Figure A2), both flux observation methods are considered sufficiently correlated in order to conduct flux partitioning in further processing. Additionally, both methods show surprisingly small offsets (−0.014 and − 0.027 for NEE) and comparable flux magnitudes (−0.34 to 0.37 and −0.27 to 0.1 g CO_2_‐C m^−2^ 30 min^−1^ for NEE). The relationships between EC and chamber fluxes could be tested positive for linearity for all parts except for NEE at the open site and *R*_eco_ at the tree site (both *p* < 0.05). In total, EC measurements tend to show higher amplitude of fluxes in both directions (uptake and emission). This shows especially for NEE and GPP at the open site and *R*_eco_ at the tree site. These differences may arise from methodological differences, caused by a general disagreement of EC and chamber measurements or by uncertainties in the partitioning of GPP and *R*_eco_, for example, NEE agrees very well at the tree site. Tests on the agreement of EC and chamber technique have so far led to different results (Lucas‐Moffat et al. 2018; Shi et al. 2022). Another source of error could be the upscaling method. EC footprints cover a larger area and can also depict parts of the site that are not represented by chamber plots.

In conclusion, it is nevertheless assumed that the systems can be compared with each other for the purposes in the presented study, as only the relation of chamber fluxes to each other is used and the general trend of fluxes agrees well.

The results of the method comparison are described and plotted in more detail in Appendix Section A.3.

### 3.4 Source Contribution

**FIGURE 4:**
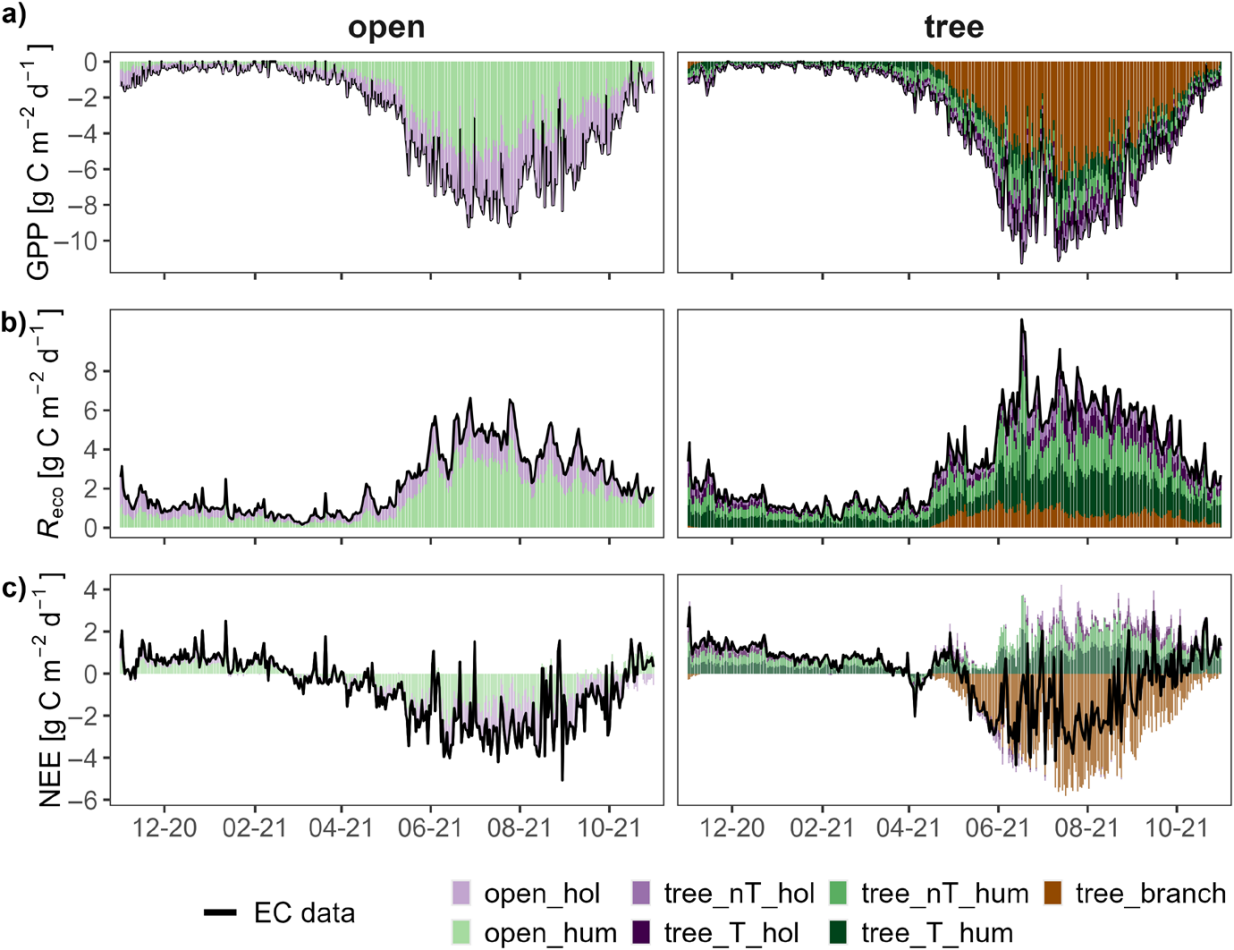
Contribution of different microforms to the total ecosystem fluxes for the open site (left) and the tree site under birch encroachment (right) derived from Eddy covariance (EC) data. ‘hum’ stands for hummock plots, ‘hol’ for hollows, ‘nT’ means that there is no tree in direct proximity and ‘T’ stands for direct proximity to trees. Each colour represents a microform; brown the birches. The black line indicates the data derived from EC. (a) GPP; (b) *R*_eco_; and (c) NEE, calculated as the difference between GPP and *R*_eco_.

Both the annual course and the magnitude of GPP were similar at the two sites. The increase in GPP started earlier (mid‐February) at the open site than at the tree site. The total GPP was slightly higher in the main vegetation period at the tree site. GPP of hummocks was higher than that of hollows at both sites and the birches dominated uptake at the tree site (Figure 4a). Highest contribution to annual GPP was calculated for the hummocks at the open site (−615.4 g C m^−2^ year^−1^), while at the tree site, the birches contribute by far the most to the annual GPP (−648 g C m^−2^ year^−1^), even though they do only contribute in the vegetation period (Table 2).

**TABLE 2:** Annual flux sums of microforms (g CO_2_‐C m^−2^ year^−1^), weighted according to their areal contribution and summed up from the source contribution. Positive values represent a release to the atmosphere and negative values mark an uptake by the vegetation. The relative share indicates the percentage contribution to the annual sum of the respective site. Water level is the mean annual water level measured for each microform on plot level.

Site	Microform	*R*_eco_	Share	GPP	Share	NEE	Water level
		g CO_2_‐C m^−2^ year^−1^	*R*_eco_ (%)	g CO_2_‐C m^−2^ year^−1^	GPP (%)	g CO_2_‐C m^−2^ year^−1^	m
Open	Hollow	296.8	36.3	−464.8	43	−168.1	−0.03
Open	Hummock	521.1	63.7	−615.4	57	−94.3	−0.13
Tree	nT_hollow	155.3	12.7	−124.8	10	30.5	−0.12
Tree	T_hollow	170.2	13.9	−112.5	9	57.7	−0.1
Tree	nT_hummock	355.7	29.1	−186	14.9	169.6	−0.23
Tree	T_hummock	401.1	32.9	−179.6	14.4	221.5	−0.27
Tree	Branch	140.7	11.5	−648	51.8	−507.3	—

Ecosystem respiration had the same annual course as GPP at both sites, but was higher at the tree site, especially during the vegetation period (Figure 4b). The total annual *R*_eco_ was more than 400 g CO_2_‐C m^−2^ higher at the tree site (809.9, respectively, 1222.8 g CO_2_‐C m^−2^ year^−1^). At both sites, hummocks accounted for the majority of the CO_2_ release. The annual sums for hummocks were 64% (open site) and 62% (tree site) of total *R*_eco_ at both sites (Table 2), even though the areal contribution is significantly lower at the open site (43.5% and 61.9%).

While the NEE proportion was more evenly split at the open site (64% hollows and 36% hummocks, close to the areal contribution given in Table A2), the data show that at the tree site mainly the trees contributed to net CO_2_ uptake, while the microforms all predominantly indicate a positive NEE (Table 2).

### 3.5 Green House Gas and Carbon Balance

Combining the CH_4_ balances derived by Welpelo et al. (2024) and the presented CO_2_ balances, full C and GHG balances were derived. The annual CH_4_ balance of the open site is 36.1 ± 3.4 g CH_4_‐C m^−2^ year^−1^, and for the tree site, it is 7.1 ± 1.5 g CH_4_‐C m^−2^ year^−1^ (Table 3).

**TABLE 3:** Annual GPP, *R*_eco_ and NEE (g CO_2_‐C m^−2^ year^−1^) derived by EC and the annual CH_4_‐C (g CH_4_‐C m^−2^ year^−1^) balance with standard deviation (SD), the annual C balance (g C m^−2^ year^−1^) and the GHG balance (t CO_2_‐eq ha^−1^ year^−1^) of the open site and the tree site (1.11.2020–31.10.2021). A global warming potential of 28 (100‐year horizon) was used for CH_4_ according to IPCC (2022).

	GPP	*R*_eco_	NEE	CH_4_‐C	Carbon	GHG
	g CO_2_‐C m^−2^ year^−1^	g CO_2_‐C m^−2^ year^−1^	g CO_2_‐C m^−2^ year^−1^	g CH_4−_C m^−2^ year^−1^	g C m^−2^ year^−1^	t CO_2_‐eq. ha year^−1^
Open	−1080.3	809.9	−262.4 ± 7.8	36.1 ± 3.4	−226.3 ± 8.5	3.38 ± 1.3
Tree	−1250.9	1222.8	−28 ± 5.1	7.1 ± 1.5	−20.9 ± 5.3	1.53 ± 0.6

Both sites showed a positive GHG balance. Due to high CH_4_ emissions, the GHG balance of the open site was slightly higher compared to the tree site, despite the high uptake of CO_2_ (3.38 ± 1.3 and 1.53 ± 0.6 t CO_2_‐eq ha^−1^ year^−1^, respectively). In contrast, both sites do show a negative annual C balance (Table 3). Due to the higher net uptake of CO_2_, the net uptake of C was more than 10 times higher at the open site than at the tree site (−226.3 ± 8.5 g C m^−2^ year^−1^, respectively, −20.9 ± 5.3 g C m^−2^ year^−1^).

## 4 Discussion

The aim of this study was to analyse the alterations in C cycling under tree encroachment. As climatic conditions, surrounding land use and land use histories are the same at the presented sites, the encroachment with birches at the tree site is suspected to be mainly a consequence of lower rewetting success and consequently more varying WL and in total drier conditions, as has been found elsewhere before (Beauregard, Lavoie, and Pellerin 2020; Holmgren et al. 2015; Murphy, Laiho, and Moore 2009). The more varying WL is the major difference apart from the trees, impacting vegetation development and increasing peat respiration. In the following, the repercussions on site level as well as on microforms and the interactions of these aspects are discussed.

### 4.1 Effects on Ecosystem Respiration and Carbon Dioxide Uptake at Site Level

The net CO_2_ uptake at the open site (−262.4 ± 7.8 g CO_2_‐C m^−2^ year^−1^) is high compared to other rewetted or undrained bog ecosystems. Similar bog ecosystems mostly exhibit a lower net uptake of CO_2_: Wilson, Farrell, et al. (2016), Lee et al. (2017) and Hurkuck, Brümmer, and Kutsch (2016) published CO_2_ uptakes ranging from −179 to –9 g CO_2_‐C m^−2^ year^−1^. There are also studies observing an uptake in the same range as the open site in some years (Korrensalo et al. 2020; Wilson et al. 2013) or even a net release of CO_2_ from rewetted peatlands (Järveoja et al. 2016; Rigney et al. 2018; Schaller, Hofer, and Klemm 2022). Tiemeyer et al. (2020) presented a German average emission factor of −40 (−240 to 130) g CO_2_‐C m^−2^ year^−1^ for rewetted organic soils, that is, a lower uptake than at the open site. However, Wilson, Blain, et al. (2016) collected annual fluxes between approximately −400 to 200 g CO_2_‐C m^−2^ year^−1^ for mean WL similar to the open site (around −0.1 to −0.15 m), which indicates the high annual and site‐specific variability of CO_2_ balances in rewetted temperate bogs. Another reason for the stronger net sink might be found in the high share of graminoids compared to other bog sites.

To our knowledge, there are no data for bogs under tree encroachment, but few data from naturally treed sites with similar balances as at the tree site: −72 and –144 g CO_2_‐C m^−2^ year^−1^ for two naturally treed bog sites in Canada (Strilesky and Humphreys 2012; Syed et al. 2006) and −53 to −73 g CO_2_‐C m^−2^ year^−1^ for a naturally wooded pre‐Alpine bog in Germany (Hommeltenberg et al. 2014). Most of the studies so far have focused on peatlands drained for forestry, which is different due to drainage history and the structured plantation of high‐yield trees. Peatlands managed for forestry often show a net uptake as a result of the high investment in biomass (Hargreaves, Milne, and Cannell 2003; Hommeltenberg et al. 2014; Lohila et al. 2011; Ojanen et al. 2019). Unfortunately, this is usually accompanied by high losses of peat C due to respiration (Mazerolle et al. 2006; Mazzola et al. 2021).

The sites differ stronger in *R*_eco_ than in GPP: In total, GPP at the open site is 86% of the tree site's GPP, while *R*_eco_ is only 66%. All seasons contributed to the total annual divergence in NEE of around 234 g CO_2_‐C m^−2^. Differences were most pronounced in summer and autumn (79 and 78.4 g CO_2_‐C m^−2^ season^−1^, respectively) when the differences in WL were also most pronounced. Continuous net uptake at the tree site is only visible during the peak vegetation period of the trees. This clearly shows the dominance of the birches in terms of the ecosystem C uptake, while the high respiration rates originate from all microforms, particularly those close to the trees. It has been shown before that increased *R*_eco_ at lowered WL is jointly caused by peat respiration and increased vascular plant growth, the latter resulting in both increased GPP and increased autotrophic respiration (Huang et al. 2021; Mezbahuddin, Grant, and Flanagan 2017; Ratcliffe et al. 2019). This indicates the higher respiration rates with almost the same GPP at the tree site—the higher production thereby is mainly determined by the periodically high uptake of the trees. Trees can, compared to mosses or grasses, have a higher CO_2_ uptake per leaf area (Heskel et al. 2013).

During the winter months, CO_2_ release is consistently higher at the tree site than at the open site. At the same time, following the dry summer of 2020, the WL was up to 0.15 m lower at the tree site. This difference can lead to high differences in CO_2_ balances (Huang et al. 2021). WL drawdown of only around 10 cm has been shown to increase by *R*_eco_ about 10%–50% (Laine, Tuittila, and Byrne 2009), which reflects the differences at our sites.

Further reasons for the higher respiration rates might be found in the dense rooting of the trees: Mazzola et al. (2021) measured increasing respiration with increasing proximity to trees and related it to higher root biomass. Rooting increases heterotrophic respiration by the aeration of the peat due to root development as well as autotrophic root respiration, especially in the vegetation period. Even if it is difficult to distinguish both processes, an increased peat respiration, and thus a release of peat‐bound C, can be assumed: Friggens et al. (2020) presented a loss of organic C accompanied by higher respiration rates in peat planted with birches compared to open bog. Available data do state that 40% and 60% of measured respiration in comparable systems as peat respiration (Hermans et al. 2022; Uri et al. 2017). Additionally, Zeh et al. (2019) showed that vascular plants can increase the C input into the peat and thus increase DOC concentrations in peat pore water. These increased root exudates can favour increased microbial activity, even if the effect is likely to be moderate (Basiliko et al. 2012; Trinder, Artz, and Johnson 2008). The stronger disturbance of the tree site is also reflected by higher DOC concentrations in the pore water, similar to Ratcliffe et al. (2017) (Table A2). Finally, the lower C:N ratio at the tree site can increase the accessibility of organic material for microbes and thus increase CO_2_ emissions as shown by Säurich et al. (2019). The low C:N ratio of the birch litter (41 ± 3), when compared to peat mosses, possibly supports this. A combination of all factors might lead to increased microbial activity at the tree site and thus higher total CO_2_ emissions.

### 4.2 Contribution of Microforms and Trees to *R*_eco_, GPP and NEE

To understand the differences between the sites, it is highly relevant to trace the CO_2_ fluxes back to their sources and sinks in the ecosystem. The main reason for a net uptake in undrained peatlands is the long‐term accumulation of dead plant material, leading to a growing peat body. In bogs, different plant functional types occur and the functional diversity can be high (Korrensalo et al. 2020). The presented sites show varying contributions of the different microforms connected to the prevailing plant functional types.

The NEE of all microforms developed towards CO_2_ release under lower WL, similar to Laine, Tuittila, and Byrne (2009). At the tree site, only the birches showed a strong net uptake, the hollows distant to a tree were close to neutral and all other microforms were net sources of C. This indicates a high loss of peat‐bound C and a missing capacity for peat formation. Consequently, C that was stored for centuries is released back to the atmosphere at that site. Munir et al. (2014) showed that especially hollows can convert from C sinks or neutrality to sources of CO_2_ under low WL. This is relevant at the tree site especially close to trees. Hollows as well as hummocks close to a tree are even a slightly larger source of CO_2_, due to higher *R*_eco_ and lower GPP. This might be caused by shading and resultant lower LAI of graminoids and mosses, disturbance and coverage by litter, as well as decreased WL and higher oxygen content in the peat due to tree root growth. This is also supported by a lower matric potential compared to the more distant hollows and their poor condition regarding *Sphagnum* growth and cover (Tables A2 and A4). Another important factor might be the increased root respiration close to trees. Generally, even though there was a short period of net uptake, neither *Eriophorum* nor *Sphagnum* could compensate for the high respiration fluxes. It might also be relevant that the richness of *Sphagnum* species is rather low with *Sphagnum cuspidatum* being the main species, which is associated with wet hollow habitats and not with drier hummocks (Clymo and Hayward 1982). As *Sphagnum* is more sensitive to dry conditions due to missing roots, it tends to be more impacted by lowered WL (Korrensalo et al. 2018; O'Neill, Tucker, and Kane 2022). In total, still, due to the higher elevation and associated thicker aerated peat layer, *R*_eco_ at hummocks overall is twice as high as at the hollows, which is also consistent with previous studies (Korrensalo et al. 2020). As the distribution of microforms can change towards a hummock‐dominated system with lower WL under tree encroachment, an increase in CO_2_ release can be expected from the microforms under these conditions (Eppinga et al. 2009). Additionally, the productivity of birches has been shown to decrease with age (Uri et al. 2017), which could also indicate a decreasing uptake by the trees in the long‐term perspective.

In comparison, both microforms at the open site do show a net uptake almost from March until mid‐October and the uptake of hummocks is more than 1.3 times higher than the uptake of the hollows. It has been shown before that hummocks have higher GPP but lower NEE due to increased *R*_eco_ (Korrensalo et al. 2020; Strack et al. 2006), which is consistent with our data. Korrensalo et al. (2020) obtained similar results in a bog in West Finland (only growing season), with all microforms showing net uptake. In other studies, GPP was the same for both microforms (Moore et al. 2002; Wu et al. 2011). Comparing the seasonal changes between the microforms at the open site, it is noticeable that the hollows are a net sink almost throughout the year, with the exception of late winter and early spring. This is probably caused by impeded photosynthesis, as hollows were inundated up to 6 cm at this time (WL separated by microforms are shown in Welpelo et al. (2024)). Kay (2019) could show that NEE could be reduced by inundation of *Sphagnum cuspidatum* . In addition, a fungus was affecting moss vitality in one plot and on site and even though conspicuous measurements were excluded (April 2021 to May 2021), there may be short‐term differences due to easy decomposable material and lowered photosynthesis. This may have reduced the role of *Sphagnum* as C sink at the open site, as they are known to take a major part in early spring when vascular plants have not yet started to photosynthesise (Korrensalo et al. 2017; Moore et al. 2006). *Sphagnum cuspidatum* is known to quickly colonise rewetted bogs and to show a high investment in biomass (Korrensalo et al. 2020; Wheeler and Shaw 1995). Highest production of biomass of *Sphagnum* was found with WL close to the surface (Korrensalo et al. 2018; Moore et al. 2002)—supporting the assumption that the conditions are more favourable at the open site.

In contrast, the hummocks were net sources of CO_2_ outside the main vegetation period as the LAI is reduced and thus the capacity for photosynthesis. In the vegetation period, hummocks with highest *Eriophorum* LAI do show the highest annual GPP, which is at both sites higher than the GPP from hollows. The seasonal change in *R*_eco_, GPP and NEE appeared to be more pronounced at hummocks compared to hollows.

### 4.3 GHG and C Balance

Although both sites have a positive GHG balance, it is 1.85 t CO_2_‐eq. ha^−1^ year^−1^ higher at the open site, which indicates a more pronounced warming effect here.

A wide range of GHG balances from rewetted bogs is available in the literature. Slightly negative (Lee et al. 2017), slightly positive (Beyer and Höper 2015; Swenson et al. 2019) and strongly positive (Schaller, Hofer, and Klemm 2022; Swenson et al. 2019) balances have been reported. Beyer and Höper (2015) presented a collection which shows a trend towards negative GHG balances with WL similar to the open site and tree site, while only mainly inundated sites had a positive GHG balance due to high CH_4_ emissions. Compared to the implied German emission factors given in Tiemeyer et al. (2020), our sites are far below all drained land uses and even below the —also positive— GHG balance for rewetted organic soils (5.5 t CO_2_eq. ha^−1^ a^−1^, including emissions from fens).

In our investigation period, CH_4_ emissions at the open site had been relatively high compared to other bogs due to periodically high WL after previous dry years (see more detailed in Welpelo et al. (2024)). In total, CH_4_ was responsible for 57% (open site) and 71% (tree site) of total CO_2_ equivalents, with a higher share at the site with lower CH_4_ emissions (Tree site). Schaller, Hofer, and Klemm (2022) stated that 78% of their GHG emissions were attributable to CH_4_. They explained their results with the short period since rewetting and expected a decrease in GHG with time—based on a frequently cited theory by Augustin and Joosten (2007), which cannot be supported by our data. Swenson et al. (2019) rather explained it as a consequence of eco‐hydrological conditions, which finally impact vegetation development—that would be supported by our sites, which had been rewetted at the same point in time. The proportion of CH_4_ emissions was relatively high, as the climatic conditions in the investigation year (high summer precipitation, low temperatures and low radiation) led to high WL, especially at the open site.

Looking at the global warming perspective and the high CH_4_ emissions, the future development of radiative forcing has to be regarded. Günther et al. (2020) modelled the impact of CH_4_ emissions emitted from wet peatlands compared to ongoing CO_2_ emissions emitted from drained peatlands on global warming. They concluded that increasing CH_4_ emissions from rewetting causes less global warming in a long‐term perspective and that rewetting all peatlands as soon as possible is necessary to keep the impact on global warming as low as possible. Mander et al. (2023) did show in their data collection that the main source of CO_2_ equivalents following all kinds of peatland restoration is CH_4_, but they also modelled the long‐term perspective (500 years), in which the GHG balance tends to become negative for almost all rewetted peatland ecosystems. This change in perspective is an important part of the ongoing discussion about peatland rewetting. One can conclude that despite the higher GHG balance at the open site, the positive effect on global warming is still greater due to the strong uptake of CO_2_.

This is also supported by the C balance, which shows a clear uptake at the open site. At both sites, CH_4_ only contributes little to the C balance (20% at the tree site and 12% at the open site). Additionally, most of the C at the tree site has been assimilated by the trees and is not saved in a long‐term C pool as in peat, as the site is too dry for the formation of (wood) peat. Instead, the site is losing peat C. Using comparable data given by Renou‐Wilson et al. (2010), the biomass gain in wood could be around 1.5 t C ha^−1^ year^−1^, of which around 25% would be stored in the belowground biomass (0.375 t C ha year). As the C is not saved and thus can be assumed to be released again in the future, wood only plays a subordinate role in the C cycling of the presented site. Furthermore, it is not harvested and stored in any long‐term wood product. Even managed tree planting on peat is very likely to result in a net loss of C (Jurasinski et al. 2024). Contrasting to that, soil mapping at the open site showed a well‐developed acrotelm, which indicates peat formation. The aim of mitigating climate change should always be to protect long‐term C pools and, if possible, to increase them (IPCC 2022).

As there is water loss due to the unintentional drainage at the tree site, some additional C export via DOC might be expected. The DOC concentrations at the tree site were higher than at the open site (Table A2), which were already slightly higher than at other successfully rewetted bogs after peat extraction (Frank et al. 2014). Still, with the roughly estimated annual water loss of around 180 to 220 mm m^−2^, DOC would add additionally around 20 to 25 g C m^−2^ to the C balance, turning the site from a slight C sink to neutral. There was no outflow expected or apparent from the open site.

We show here the opportunity of reaching a C sink and thus peat growth in the long‐term. Furthermore, the results emphasise the importance of full rewetting to reach these favourable conditions. We could demonstrate that in contrast tree encroachment as a consequence of inadequate rewetting can increase respiration rates rather than increase the GPP of a bog. Although total GHG emissions are slightly higher from the open site, creating C sinks is preferable from a long‐term perspective. Thus, our results advocate for improving rewetting of incompletely rewetted bogs and preventing or reducing tree encroachment.

## Author Contributions

**Carla Welpelo:** data curation (lead), formal analysis (lead), investigation (equal), methodology (equal), visualization (lead), writing – original draft (lead), writing – review and editing (lead). **Maren Dubbert:** conceptualization (equal), funding acquisition (equal), methodology (equal), project administration (equal), resources (equal), supervision (equal), writing – review and editing (equal). **Bärbel Tiemeyer:** conceptualization (equal), methodology (equal), project administration (equal), resources (equal), supervision (equal), writing – review and editing (equal). **Alexander Knohl:** methodology (equal), supervision (equal), writing – review and editing (equal). **Arndt Piayda:** conceptualization (equal), data curation (supporting), funding acquisition (equal), methodology (equal), project administration (equal), resources (equal), supervision (equal), validation (equal), writing – original draft (supporting), writing – review and editing (equal).

## Conflicts of Interest

The authors declare no conflicts of interest.

## Data Availability

Data are available at https://doi.org/10.3220/DATA20241107163353‐0.

## Appendix A

### A.1 Further Site‐Specific Environmental Conditions

**TABLE A1:** Peat properties for the peat horizons in the first meter of both sites. Von Post is the decomposition rate after von Post (1924), TN, total nitrogen; TC, total carbon. pH is measured in a CaCl_2_ solution, P and Fe are extracted using a calcium acetate lactate extract (_cal_).

Location	Horizon depth	von Post	TN	TC	C/N	pH (CaCl_2_)	P_CAL_	Fe_CAL_
	(cm)		(g kg^−1^)	(g kg^−1^)			(mg kg^−1^)	(mg kg^−1^)
Open site	0–11	H2	8.3	506.0	61	3.3	61.6	25.0
	11–56	H3	6.8	514.9	75	3.2	37.2	13.4
	56–68	H4	10.8	548.5	51	2.9	4.9	95.8
	68–92	H5	8.9	533.6	60	3.0	3.2	44.2
	92–110	H5	8.7	537.9	62	3.1	2.1	25.8
Tree site	0–30	H2	12.3	510.1	42	3.2	124.3	21.7
	30–78	H7	12.4	550.5	45	3.0	5.5	95.7
	78–100	H8	17.2	593.6	34	3.1	1.5	81.2

**TABLE A2:** Areal contribution, water level, leaf area index (LAI), matric potential and concentrations of dissolved organic carbon (DOC) in the pore water (30 cm depth) for the different microforms.

Microform	Areal contribution	Water level (mean ± range)	Vasc. LAI (mean)	Moss LAI	Matric potential (mean)	DOC
	(%)	(m)	(m^2^ m^−2^)	(m^2^ m^−2^)	(kPa)	(mg L^−1^)
open_hollow	56.5 ± 2.2	−0.03 (−0.18 to 0.06)	0.2 ± 0.1	22 ± 7.2	0.8 ± 0.3	66 ± 3
open_hummock	43.5 ± 2.2	−0.13 (−0.30 to −0.01)	5.5 ± 3.3	5.1 ± 0.7	−1.2 ± 0.7	65 ± 4
tree_nT_hollow	19.3 ± 12.2	−0.12 (−0.30 to 0.04)	0.6 ± 0.4	14 ± 4.6	−0.6 ± 0.8	105 ± 21
tree_nT_hummock	31.2 ± 15.1	−0.23 (−0.42 to −0.09)	4.7 ± 2.2	2.1 ± 2	−2.3 ± 0.8	106 ± 21
tree_T_hollow	18.8 ± 6.3	−0.1 (−0.29 to 0.05)	0.8 ± 0.6	8.1 ± 2.3	−2 ± 0.9	115 ± 31
tree_T_hummock	30.7 ± 12.9	−0.27 (−0.45 to −0.1)	2.9 ± 1.4	0.7 ± 0.7	−2 ± 0.8	123 ± 22

### A.2 Data Processing of CO_2_ Flux Measurements

#### A.2.1. Data Processing—Eddy Covariance

Half‐hourly CO_2_ fluxes from high frequency raw data were calculated using the Advanced Mode of the processing software EddyPro (LI‐COR, Version 7.0.9). Standardized settings for calculations and corrections chosen for application in EddyPro are summarised in Table A3.

**TABLE A3:** Settings in EddyPro (Version 7.0.9; LI‐COR Environmental) for flux calculation (Advanced mode).

Processing step	Open site	Tree site
Analyzer	LI‐7500‐A; open path	Li‐7200; enclosed path
Axis rotation for tilt correction	Double rotation (Wilczak et al. 2001)	
Detrending method	Block average (Gash and Culf 1996)	
Time lag detection and compensation	Covariance maximization with default (LI‐COR 2022)	
Compensation for air density fluctuation	Webb‐Pearman‐Leuning (Webb et al. 1980)	—
Low frequency range correction	Analytic correction of high‐pass filtering effects (Moncrieff et al. 2004)	
High frequency range	Correction of low‐pass filtering (Moncrieff et al. 1997)	

The full output of EddyPro was used for further data processing. The CO_2_ flux data was filtered as follows: Only data with quality check flag 0 (good quality) and 1 (data quality ok) were kept (Mauder and Foken 2004). Data with a signal strength below 50% were set to NA and fluxes with CO_2_ variance > 400 were discarded. To limit the output to the targeted footprint (FP), the FP data provided by EddyPro, calculated according to Kljun et al. (2004), was used. All datapoints with their peak flux source outside of the target area were removed. For the open path, additional data filtering was applied: To prevent incorrect data caused by dust or water on the lens, all data points with a RH > 97% and concurrent precipitation were removed (eliminating 261 data points, 1.5%). The selected threshold for differentiating between daytime and night‐time data is set to 20 W m^−2^ global radiation (Rg) for all processing steps.

The remaining data was tested for outliers following Papale et al. (2006), using 13 day blocks and a z value of 7. At the tree site, 210 (1.2%) datapoints were removed as outliers, at the open site 955 (5.5%).

The R package REddyProc (Wutzler et al. 2022) was used to calculate friction velocity (u*) thresholds and to conduct gapfilling and flux partitioning. To determine and exclude data from low turbulence conditions, estimation of u* thresholds was performed using ‘EstimateUstarScenarios’, which is determining individual u* for each season using bootstrap scenarios (Wutzler et al. 2018).

In total, 43% of the original data remained from the tree site and 55% from the open site, respectively.

Gapfilling was done by the commonly used marginal distribution sampling (MDS), which is a combination of the look up table approach and the mean diurnal circles method as described in Reichstein, Kätterer, et al. (2005). As environmental parameters, vapor pressure deficit (VPD, margin 5 hPa), *T*_soil_ in 5 cm depth (margin 2.5°C) and Rg (margin 50 W m^−2^) were used. To partition NEE into ecosystem respiration (*R*_eco_) and gross primary production (GPP), night‐time partitioning was applied as described in Reichstein, Falge, et al. (2005). Standard deviation of annual NEE was calculated as described in Pastorello et al. (2020) for the remaining original data and derived from the REddyProc output for the gapfilled datapoints. The final footprint (Figure 1) was calculated using the model presented by Kljun et al. (2015) using the available R code. If GPP was accidently modelled as positive, it was set to zero and the difference was added to *R*_eco_.

Energy balance closure (EBC) was calculated using half‐hourly daytime data. The EBC was 76% on the open site and 83% on the tree site, respectively.

#### A.2.2. Data Processing and Calculation of Annual Balances—Chamber Measurements

Fluxes were calculated using a linear regression inside a moving window. Depending on solar declination, the length of the moving window ranged from 40 (summer) to 60 s (winter). No flux was calculated for any window if ΔPAR > 15% (except if PAR >150 W m^−2^) or Δ*T*_chamber_ > 1.5°C. For each measurement, the window with the highest *R* ^2^ was chosen for the final flux calculation. If *R* ^2^ was over 0.9 and Δ*T*_chamber_ > 1.5°C, the flux was manually checked and kept if no change in slope was detectable. Additionally, all fluxes below the Minimal Detectable Flux (MDF) as described in Fiedler et al. (2022) were set to zero (*n* = 46).

Illogical high morning fluxes were identified if all the following criteria met at plot level: Earliest *R*_eco_ flux of the day, measured before 9 a.m., highest *R*_eco_ and the highest start concentration (ppm) of CO_2_ of the respective day. Fluxes were removed from the data set (*n* = 60). Overall, 3102 fluxes remained for further analyses.

Daily fluxes for each microform were calculated in a campaign based approach to account for seasonal succession of biomass (Leiber‐Sauheitl et al. 2014; Oestmann et al. 2022). To increase the robustness of the fit of the response functions, up to three campaigns were pooled. For *R*_eco_, the Arrhenius response function (Lloyd and Taylor 1994) was used (Equation 1).

(1)
$$R_{\mathrm{eco}}\left(T\right)=R_{\mathrm{ref}}\times \exp\left[E_{0}\times \left(\frac{1}{T_{\mathrm{ref}}-T_{0}}-\frac{1}{T_{\text{soil}}-T_{0}}\right)\right]$$

With *R*_ref_ being the respiration rate at a reference temperature (mg m^−2^ h^−1^), *E*_0_ activation‐like parameter (°K) and *T*_soil_ the soil temperature in 5 cm depth (K). The reference temperature (*T*_ref_) is set to 283.15 K and *T*_0_ to 227.13 K. If changes of *T*_soil_ during the campaign were ≤ 2°C, *R*_ref_ was set to the median of all *R*_eco_ fluxes.

Using *T*_soil_ from each microform, *R*_eco_ was calculated on a half‐hourly basis using the derived values of *e*_0_ and *R*_Ref_, building a distance‐weighted average flux using parameters from the previous and subsequent campaign. GPP was calculated subtracting the modelled *R*_eco_ from the measured NEE. In the next step, GPP was modelled using data for half‐hourly data using a light response curve based on the Michaelis–Menten kinetics model (Falge et al. 2001) (Equation 2).

(2)
$$\mathrm{GPP}\left(\mathrm{PAR}\right)=\frac{\mathrm{GPP}2000\times \alpha \times \mathrm{PAR}}{\mathrm{GPP}2000+\alpha \times \mathrm{PAR}-\frac{\mathrm{GPP}2000}{2000\;\text{μmol}\;m^{-2}s^{-1}\;}\times \mathrm{PAR}}$$

where PAR describes the photon flux density (μmol m^−2^ s^−1^), GPP2000 the carbon fixation rate (mg CO_2_‐C m^−2^ h^−1^) at a PAR of 2000 μmol m^−2^ s^−1^ and *α* (mg CO_2_‐C m^−2^ h^−1^/μmol m^−2^ s^−1^) the initial slope of the light response curve (Falge et al. 2001). Annual time series were then interpolated following the method used for *R*_eco_, using half‐hourly PAR values. To complete NEE time series, derived GPP and *R*_eco_ values were added together.

Annual balances and uncertainties were estimated by a bootstrap approach. Therefore, resampled campaigns of GPP and *R*_eco_ were fitted again with their response functions. Standard errors were calculated from these bootstrapped fits (*n* = 1000).

A more detailed description of the calculations is available in Oestmann et al. (2022).

### A.3 Method Compatibility

**TABLE A4:** Vegetation survey of each plot at the field sites in Lichtenmoor (2020‐08‐26). Numbers denote coverage in %. Vegetation survey was supported by Amanda Grobe.

Plot	Open						tree_nT						tree_T					
	hol_1	hum_1	hol_2	hum_2	hol_3	hum_3	hol_1	hum_1	hol_2	hum_2	hol_3	hum_3	hol_1	hum_1	hol_2	hum_2	hol_3	hum_3
Total	80	100	100	30	100	100	99	80	95	100	100	100	50	90	90	45	80	45
Vascular plants	4	75	5	25	3	20	5	70	8	80	15	75	1	60	30	40	1	45
Mosses	80	50	100	15	100	80	99	50	95	75	90	60	50	10	80	20	80	25
Peat								2	5				2			25		
Water																		
Litter	25	20	3	70	1	20	2	20	1	20	10	25	30	40	15	30	20	55
Mosses																		
*Sphagnum cuspidatum*	80	50	100	15	100	80	97	5	94		90	60	50	1	65		80	5
*Sphagnum fallax*											1							
*Sphagnum fimbriatum*									1	73			1			1		5
*Sphagnum palustre*														4				
Liverwort										1								
Other mosses						1	2	45	1	1	5	1	20	5	15	20	0.5	15
Vascular plants																		
*Betula pubescens*						1	4		1			0.5	0.5	1	1			
*Dryopteris carthusiana*						0.5		10			0.5		0.5	10		1		5
*Eriophorum angustifolium*	4		5		2												0.5	
*Eriophorum vaginatum*		75	0.5	25	1	20	1	60	8	80	5	8	0.5	50	30	40	0.5	40
*Pinus sylvestris*		0.5																
Seedlings												0.5					0.5	
Mushrooms																		
Small mushroom	1		3		1													
